# Linalool Exhibits Cytotoxic Effects by Activating Antitumor Immunity

**DOI:** 10.3390/molecules19056694

**Published:** 2014-05-22

**Authors:** Mei-Yin Chang, Yi-Ling Shen

**Affiliations:** Department of Medical Laboratory Science and Biotechnology, School of Medical and Health Sciences, Fooyin University, Ta-Liao District, Kaohsiung 83102, Taiwan; E-Mail: wind0935@hotmail.com

**Keywords:** linalool, *p*-coumaric acid, cell apoptosis, cell cycle, cytokine array

## Abstract

According to recent studies, the *Plantaginaceae*, which are traditional Chinese herbal remedies, have potential for use in viral infection treatment and cancer therapy. Linalool and *p*-coumaric acid are two of the biologically active compounds that can be isolated from the *Plantaginaceae.* This study mainly focused on investigating the bioactivity of linalool as well as the bioactivity of *p-*coumaric acid in terms of their cytotoxic effects on cancer cells. Whether the mechanisms of such effects are generated through apoptosis and immunoregulatory activity were also investigated. By using WST-1 analysis, it was shown that linalool and *p*-coumaric acid have good inhibitory effects against breast, colorectal and liver cancer cells. The IC_50_ values of linalool for those cancer cell types were 224 μM, 222 μM, and 290 μM, respectively, and the IC_50_ values of *p*-coumaric acid were 693 μM, 215 μM and 87 μM, respectively. Cell cycle analysis also confirmed that linalool and *p*-coumaric acid can lead to apoptosis. By using flow cytometry, it was determined that treatment with linalool rather than *p*-coumaric acid significantly increased the sub-G1 phase and that there were more cells concentrated in the G1 phase. Furthermore, by using cytokine array analysis, we found that linalool can stimulate IFN-γ, IL-13, IL-2, IL-21, IL-21R, IL-4, IL-6sR and TNF-α secretion. This demonstrated that in addition to the bidirectional regulation capabilities found in linalool, it also induces Th1 cellular immune response in T-47D cells. These results showed that linalool holds great potential for use in cancer therapy, and we believe that it could provide an alternative way to take action against tumors.

## 1. Introduction

According to statistics from the Ministry of Health and Welfare, cancer has been the top cause of death in Taiwan for the past 30 years, and the trend is rising every year. On average, 120 persons will be killed by cancer each day [1]. In terms of the worldwide incidence rates of cancers, the most commonly occurring cancers in men are lung cancer, colorectal cancer and liver cancer, while the most common cancers in women are breast cancer, colorectal cancer, lung cancer and liver cancer [2]. Generally speaking, breast cancer is the most common cancer among women, and lung cancer is the most common cancer in men.

The formation of cancer can be largely affected by factors such as genes, viruses, chemicals and radiation, but the loss of immunity is also closely linked with cancers. As the immune system plays a part during the recovery from cancers or viral infections, it is also an important preventive and therapeutic measure to regulate the immune system and strengthen its immunity to provide the body with a normal defense mechanism for lowering the occurrence of such illnesses. From an immunological standpoint, the monitoring of the immune system relies on the prevention of infectious diseases as well as the formation of tumors. As the immune system begins its defensive role after the infection of viruses as well as before the formation of tumors, it therefore plays a crucial role in suppressing the occurrence of infectious diseases, as well as suppressing the formation of tumors and aiding in their removal [3,4].

By extracting constituents from many specimens of plant, various kinds of active constituents can be found. For instance, oridonin and ponicidin separated from *Rabdosia rubescens* L. are capable of suppressing cell proliferation and stopping the progression to the G2 and M phase by inducing the arrest of the G1 phase [5]. 4-Shogaol separated from dried red ginger has the ability to suppress the migration and invasion of breast cancer cells (MDA-MB-231) [6]. Polysaccharides isolated from *Bupleurum falcatum* L. can be applied for stimulating the growth of B cells as well as fighting gastritis [7]. Among natural products betulinic acid, α-amyrin acetate, lupeol acetate, oleanolic acid, ursolic acid and their derivatives have shown interesting potential analgesic and anti-inflammatory activity [8]. Liu had proven through animal testing that triterpenoids possess anti-inflammatory and anti-hyperlipidemic effects [9]. Inada *et al.* had discovered that triterpenoids have the characteristic of suppressing TPA-induced EBV early antigens (EA) in cells [10], and Constantinou *et al.* have highlighted in reports that flavonoids in plants manage to suppress the activity of enzymes including protein kinase C and DNA topoisomerase II (topo II) [11]. Yu *et al.* had suggested in studies that using glycosidase to catalyze *Scutellaria* *baicalensis* L. offers a promising approach for increasing its anticancer activity [12]. In 1998, Nishioka *et al.* discovered that baicalein and baicalin can be used to stop the intake of carbohydrates through the suppression of α-glucosidase, thereby suppressing sugar levels after meals [13]. Ortiz de Urbina *et al.* believed that aucubin in iridoid contained activity that can be used for anti-inflammatory purposes as well as for relieving spasms [14]. Stigmasterol separated from *Liriodendron tulipifera* inhibits tyrosinase activity and reduces the melanin content in animal cells [15]. Subamolide E separated from *Cinnamomum subavenium* causes DNA damage in the sub-G1 phase. In addition, it also possesses antimigratory activities [16].

**Figure 1:**
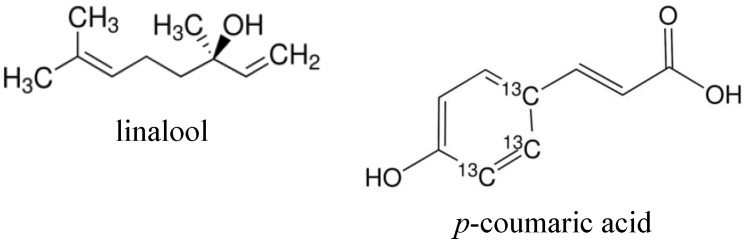
The chemical structures of linalool and *p*-coumaric acid.

According to the aforementioned references, there are multiple aspects to the medicinal activities of natural plants. Although there are already various drugs available for the treatment of both cancers and viral infections, it remains necessary that effective suppressive drugs continue to be developed because of the toxicities, side-effects, and drug resistance increase of existing treatments [15,17,18]. Therefore, the use of natural plants with multiple medicinal activity aspects may present new opportunities. For the past 10 years, extensive studies have been carried out on the activity of plants from the *Plantaginaceae* (*Plantago major* L. and *Plantago asiatica* L.) as well as their constituents [19,20,21,22], and further investigations of the bioactivities and mechanisms of action of the monoterpenoid linalool as well as *p-*coumaric acid (Figure 1) will be described herein.

## 2. Results and Discussion

### 2.1. WST-1 Assay Analysis of the Suppression of Cancer Cell Growth with Constituents

WST-1 assay analysis was conducted to test for the cytotoxic effects of linalool and *p-*coumaric acid compared with the control (5-fluorouracil; 5-FU) on A549, T-47D, SW 620 and Hep G2 cells after 48 h. The experimental results indicated that both linalool and *p*-coumaric acid can effectively suppress the growth of cancer cells with IC_50_ values ranging from 87–693 μM (Table 1). Not only did the monoterpenoid linalool manage to suppress SW 620, T-47D, Hep G2 and A549 cells with IC_50_ values of 222, 224, 290 and 438 μM, respectively, but a dose response was also exhibited while suppressing SW 620 and Hep G2 cells (data not shown). The phenolic compound *p-*coumaric acid was also capable of suppressing SW 620, Hep G2, A549 and T-47D cells with IC_50_ values of 87, 215, 412 and 474 μM respectively. In addition, dose dependence was exhibited in its suppression of SW 620 and Hep G2 cells (data not shown). Taken together, the results above indicate that both linalool and *p-*coumaric acid inhibit activities that was capable of suppressing the growth of cancer cells. Compared with the 5-FU control, the suppression of T-47D cell by linalool and *p-*coumaric acid, and the suppression of Hep-G2 by *p-*coumaric acid had a statistically significant difference (*p* < 0.05) (Table 1).

**Table 1:** The cytotoxic effect of linalool and *p*-coumaric acid determined by cytotoxicity assay.

Cell Line	50% Inhibitive Dose (IC50: μM)		
	5-Fluorouracil	Linalool	*p*-Coumaric Acid
SW 620	78 ± 6.60	222 ± 5.44	87 ± 6.33
Hep G2	278 ± 19.40	290 ± 5.31	215 ± 6.58 *
A549	45 ± 2.50	438 ± 6.48	412 ± 5.50
T-47D	648 ± 36.20	224 ± 4.40 **	474 ± 12.10 *

Data are presented as mean ± S.D, *n* = 3. Different letter notations indicate the statistical significance between control (5-Fluorouracil) and constituent treatment groups. * *p* < 0.05 and ** *p* < 0.001 against control,respectively.

**Figure 2:**
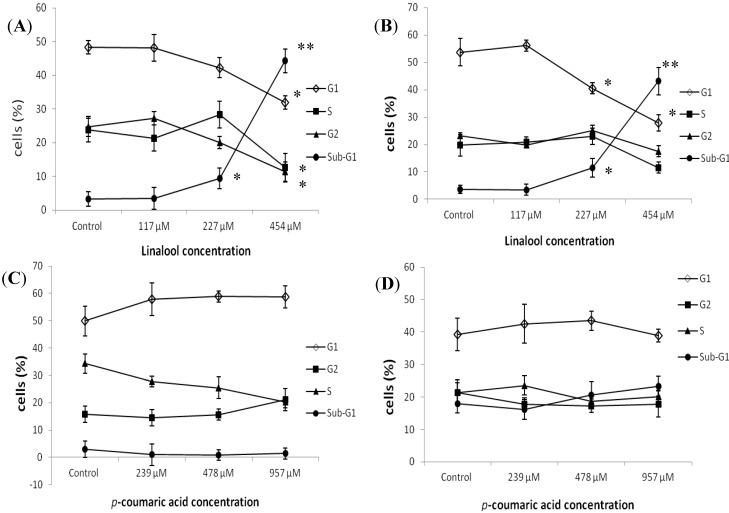
Cell cycle distributions of linalool-treated and *p-*coumaric acid-treated T-47D cells were analyzed by flow cytometry at 24 h and 48 h. The percentage of the total cells that were in the sub-G1 phase was calculated expressed on each histogram. (**A**) The concentrations of linalool were 117, 227, and 454 μM for 24 h; (**B**) The concentrations of linalool were 117, 227, and 454 μM for 48 h; (**C**) The concentrations of *p-*coumaric acid were 239, 478, and 957 μM for 24 h; (**D**) The concentrations of *p-*coumaric acid were 239, 478 and 957 μM for 48 h.Data shown are mean ± S.D. for the three independent experiment results. * *p* < 0.05 and ** *p* < 0.001 against control (0.1%; ethylalcohol), respectively.

### 2.2. Flow Cytometry Analysis of the Effects of Constituents on Cell Cycle Distribution

Using flow cytometry for the analysis of cell cycle distribution, it could be determined that the cells were undergoing apoptosis if a peak is recorded before the G_1_ phase. Typically, in addition to representing the ratio of cells migrating towards apoptosis, the sub-G_1_ phase also represents the level of DNA damage. After treating SW 620, Hep G2 and A549 cells with linalool and *p-*coumaric acid for 24 and 48 h, respectively, no significant sub-G_1_ phase was generated (data not shown). After T-47D cells were treated with linalool for 24 h, the sub-G_1_ phase percentages at 0, 117, 227, and 454 μM were 3.59%, 3.48%, 11.56%, and 43.14% respectively, indicating that the ratio that the sub-G_1_ phase occupies increases as the concentration increases (Figure 2A). After T-47D cells were treated with linalool for 48 h, the sub-G_1_ phase percentages resulting from 0, 117, 227, and 454 μM were 3.22%, 3.48%, 9.41%, and 44.3% respectively, thus indicating that the ratio that the sub-G_1_ phase occupies increases as the concentration increases (Figure 2B). For the *p-*coumaric acid treatment of T-47D cells for 24 and 48 h, however, no significant sub-G_1_ phase was generated (Figure 2C,D). As shown in the quantitative graph, the ratio that the sub-G_1_ phase occupied was not significant as the concentration increased.

### 2.3. Cell Migrations of Ductal Breast Epithelial Tumor by Constituents

**Figure 3:**
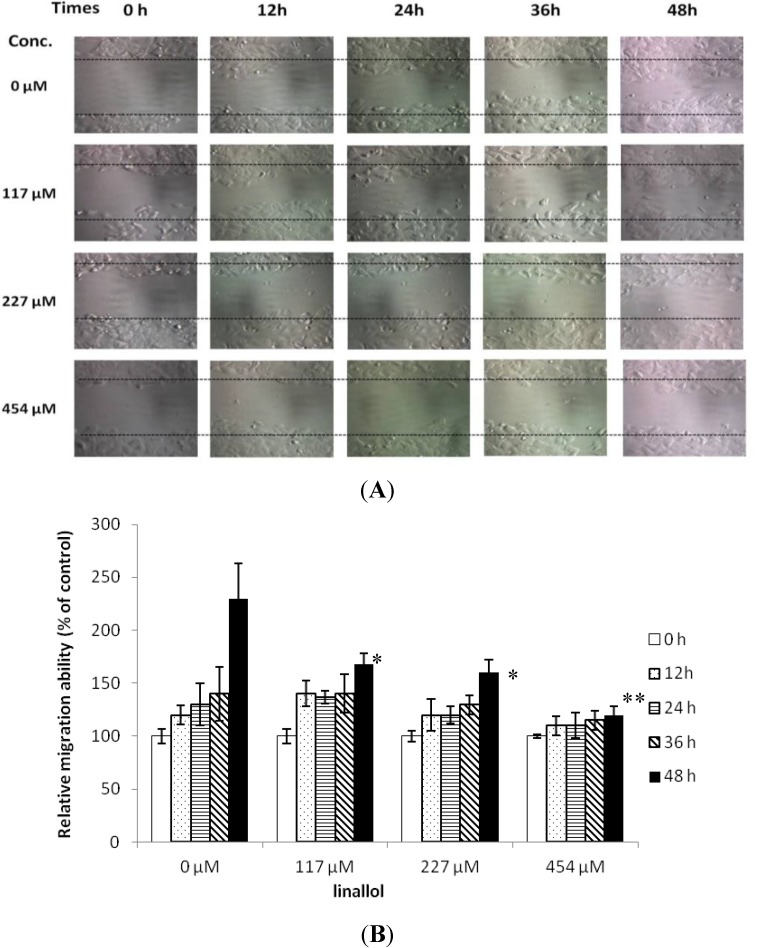
*In vitro* wound-healing assay on linalool-treated breast epithelial tumor cell: (**A**) migratory photographs of T-47D cells trated with PBS (control: linalool 0 μM) and linalool at 117, 227 and 454 μM for 12, 24, 36 and 48 h; (**B**) quantification of the migration of linalool or PBS-treated cells. * *p* < 0.05 and ** *p* < 0.001 against control, respectively.

To determine the effects of linalool on T-47D cell migration, an *in vitro* wound healing assay was performed. The result showed that cell migration was affected while treated with linalool at 117, 227 and 454 μM for 12, 24, 36 and 48 h (Figure 3), and the significant difference (*p* < 0.05) revealed while treated for 48 h at each treated concentration compared with the control (linalool 0 μM).

### 2.4. Cytokine Array Analysis of the Effects of Constituents on the Cytokine Secretion

**Figure 4:**
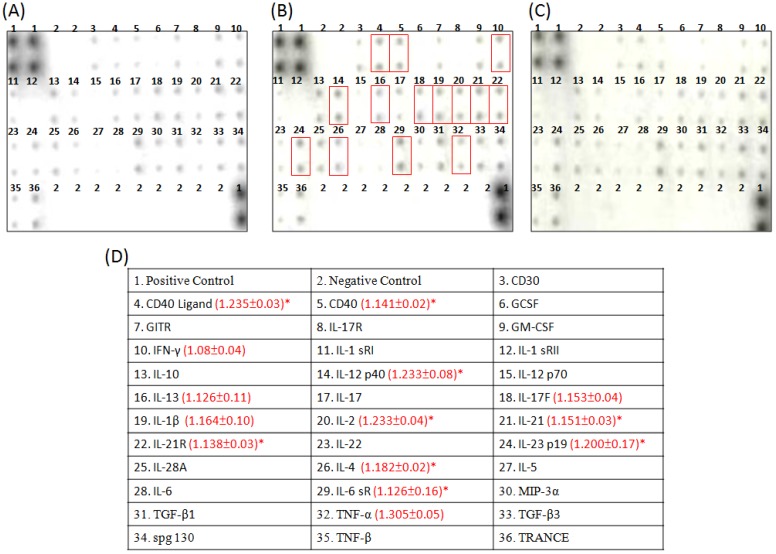
Images from the cytokine array assay. Lymphocytes were incubated with linalool and *p*-coumaric acid at 227 μM and 478 μM, respectively, for 24 h, and then the cytokine secreted was measured by cytokine array assay. (**A**) control (unstimulated); (**B**) linalool (227 μM) at 24 h; (**C**) *p-*coumaric acid (478 μM) at 24 h; (**D**) positions of cytokine probes on membrane array and the mean density of each differentially secreted probes for linalool (*n* = 3) which was divided by the mean density of unstimulated control. * *p* < 0.05 and ** *p* < 0.001 against control, respectively.

From the results of the cell cycle analysis, it was found that linalool was capable of promoting the apoptosis of T-47D cells. This study completed a cytokine array analysis on the cytokines secreted from the stimulation of lymphocyte with linalool to understand the related linalool-induced immune mechanisms (Figure 4). As shown in Figure 4B, linalool could stimulate lymphocyte in its production of CD40 Ligand, CD40, IFN-γ, IL-12 p40, IL-13, IL-17F, IL-1β, IL-2, IL-21, IL-21R, IL-23p19, IL-4, IL-6sR and TNF-α. In comparison, stimulation of the production of cytokines with *p-*coumaric acid was not as significant (Figure 4C).

As the research and development of clinical drugs with natural herbs by the National Cancer Institute (NCI) during the end of the 1950s had yielded promising results, many compounds extracted from Traditional Chinese Medicines were found to possess anti-tumor activities and the results of these natural plant studies have been widely applied [23,24,25,26,27]. For its fragrance, the world had been using more than 1,000 tons of linalool every year, most commonly by the perfume industry as well as a form of traditional medicine. In the perfume industry, linalool is added in products such as cosmetics, perfumes, shampoos, soaps, and also in detergents as well as other cleaning agents [28]. For the traditional medicine, during early forms of therapy, linalool was used in alleviating pain caused by acute and chronic diseases or as a suppressant for *Candida albicans*, *Escherichia coli*, and *Staphylococcus aureus*[29]. As highlighted by earlier references, linalool inhabits analgesic and anti-inflammatory properties that can be used to suppress cholinesterase as well as hyperplasia. Inflammatory reactions, caused by diseases such as diabetes and cardiovascular disease, can be alleviated through the use of linalool [30,31]. Animal tests had also proved that dosage effects were exhibited when linalool was used in the alleviation of effects in the central nervous system. References also demonstrated that linalool could be combined into N-methyl-D-aspartate (NMDA) to achieve analgesic purposes [32]. According to the cell growth cycle and prior research, the cytotoxicity times can be 24, 48, 72, and 96 h [33,34]. This study analyzed the cytotoxicity of linalool and p-coumaric acid and their effects on the cell cycle at 24 h and 48 h. Linalool showed a good cytotoxic activity against T-47D cells with an IC_50_ value of 224 μM, compared with a value of 648 μM for 5-FU (Table 1). Based on DNA decomposition and the accumulated phenomena of the sub-G1 phase, linalool may be thought to suppress T-47D cells by inducing cancer cells to undergo apoptosis, thus resulting in cell death. Linalool was also demonstrated to affect the cell migration on T-47D cell. Linalool thus presents tremendous potential for treating cancer.

The phenolic *p-*coumaric acid is commonly found in many fruits and vegetables, including apples, pears, beans and teas. Phenolic compounds have anti-inflammatory, anti-oxidiants, anti-HIV and cytotoxic effects activities [35,36]; *p-*coumaric acid can also suppress the formation of the carcinogen nitrosamine to lower the risk of stomach cancers through its anti-oxidant mechanism. In addition, *in vitro* tests conducted by Kong *et al.* have also proved that *p-*coumaric acid could be used as a tyrosinase inhibitor for suppressing the biosynthesis of melanin, thereby suppressing the growth of Melanin tumors [35]. Other studies had also proved that *p-*coumaric acid could regulate and control the MYC, CDKN1A, PCNA, CDC25A, ODC1, CCNA2 and CCNB1 genes in the cell of colorectal cancer, causing an effect in its cell cycle and delayed the G2/M phase [37]. Furthermore, animal tests conducted by Scheepens *et al.* had proved that *p-*coumaric acid would have anti-depressing activities at the concentration of 3–90 mg/kg; and even when the rats ingested *p-*coumaric acid, with an LD_50_ of 2,850 mg/kg, no toxic reactions were exhibited. From the abovementioned results, it can be deduced that *p-*coumaric acid not only has a wide range of activities, but it is also a safe product [38]. The results from this study indicated that in addition to the suppression of colorectal cancer, *p-*coumaric acid can concurrently suppress lung cancer, breast cancer and liver cancer cell lines (Table 1). However, the cytotoxic effects on suppressing cancer cells with *p-*coumaric acid in this study was not by affecting the cell cycle but via some other pathways. This is worthy of future investigation.

Immune activation is capable of promoting the hyperplasia of lymphocytes and stimulating the secretion of cytokines. Through the secretion of cytokines as well as other forms of immune cell communication and regulation, such as the activation of macrophages for producing cytotoxic agents, like nitric oxide, TNF-α and IFN-γ secretion, it can protect the host against the development of diseases and tumors, and remove any damages caused by any abnormal cells or infectious diseases [39]. While eliminating the immune response of tumors, the effects of the tumor can be directly eliminated via CTL or NK cells of lymphocytes, or cytokines that are released after the activation of the Th cells. Cytokines is also capable of stimulating the cytotoxicity of the LAK cells or promoting the antibody-dependent cell-mediated cytotoxicity (ADCC) [40]. The results from the cytokine array of this study also proved that linalool is capable of stimulating the secretion of CD40 Ligand, CD40, IFN-γ, IL-12 p40, IL-13, IL-17F, IL-1β, IL-2, IL-21, IL-21R, IL-23 p19, IL-4, IL-6 sR and TNF-α (Figure 3B). It is discovered that IFN-γ, IL-2 and TNF-α are related to the cellular immune, thereby indicating that not only does linalool have bidirectional regulation capabilities, it also induced T-47D cells into the cellular immune response of Th1 cells.

The results obtained in this study demonstrate that linalool can have cytotoxic effects by inducing cells to undergo apoptosis, triggering cell death. The majority of studies pertaining to the monoterpenoid linalool have typically focused on its capacity to suppress microorganisms, with few having endeavored to research aspects of its anti-cancer activity. Linalool offers tremendous potential in terms of treating cancer and immunity. We believe that this substance can substantially enhance tumor treatments and provide novel starting points for future anti-cancer research.

## 3. Experimental Section

### 3.1. Cytotoxic Effects of Constituents

SW 620, T-47D, A549 and Hep G2 were counted and seeded on culture plates with 1 × 105 cells/mL and treated with different concentrations of linalool (molecular weight 154.25; Sigma Chemical Co., St. Louis, MO, USA) and *p*-coumaric acid (molecular weight 164.16; Sigma Chemical Co.), respectively. For each 96-well plate, a blank-cell (unstimulated) control group and a standard 5-Fluorouracil (molecular weight 130.08, Sigma Chemical Co.) control group were prepared. Linalool and *p*-coumaric acid were dissolved in ethyl alcohol at a stock concentration of 6.48 M and 6.09 M, respectively, and stored at −20 °C. 5-Fluorouracil was dissolved in dimethyl sulfoxide (DMSO) at a stock concentration of 6.69 M and stored at −20 °C. For *in vitro* use, linalool, *p*-coumaric acid and 5-fluorouracil were diluted in culture medium. An aliquot was used only once. Triplicates of each treatment were used. The samples were placed in 96-well plates and cultivated in an incubator at 37 °C with 5% CO_2_ for 24 h. Subsequently, a WST-1 mixture (Roche Diagnostics GmbH, Mannheim, Germany) was introduced into the incubator. Following 2 to 4 h reaction time, an ELISA reader (Multiskan EX, Labsystems, Stockholm, Sweden) was used to test the light absorption values, which were 450 nm. The suppression rate for cell death by drug was calculated as follows:

Inhibition (%) = [100 − (OD_T_/OD_C_ × 100)] %

OD_C_: The light absorption rate of the control group without test drug
OD_T_: The light absorption rate of the various experimental groups administered with varying concentrations of the test drug
IC_50_: Concentrations were compared and tested to achieve the optimal concentration for a 50.0% cell death rate.

### 3.2. Flow Cytometry Analysis

The cell samples at different concentrations were placed in 6-well plates. Different concentrations of linalool and *p*-coumaric acid were applied to the cells. Subsequently, ethyl alcohol (final concentration = 0.1%) was used as the control group. The cells were collected after 6 h and secured with 99.9% alcohol. Then, phosphate buffered saline (PBS; Thermo Scientific, Rockford, IL, USA), RNase (10.0 µg/mL; Sigma Chemical Co.), and Triton (0.5%; Sigma Chemical Co.) were administered separately (dark operations). The supernatant was removed from the solution following centrifugation, and propidium iodide (PI; 0.5%; Sigma Chemical Co.) dye was administered. Then, the contrast solution was again centrifuged and the subsequent supernatant was removed. Finally, PBS was administered to wash and disperse the solution. The solution was then passed through a 40-µm mesh filter into a test tube. The flow cytometry analysis (FACSCalibur, Becton Dickinson, Franklin Lakes, NJ, USA) method was employed to analyze 10,000 cells [41].

### 3.3. Wound Healing Migration Assays

The cellular migration potential was determined by wound healing migration assays, which was performed according to the reported methods [16]. First, 5 × 10^5^ cells were cultured in 12-well plates, and grown to complete confluence. The 200 μL plastic pipette tip was used perpendicularly to the plate to create cell wounds with consistent space between each wound. Then the cell culture medium was removed and PBS was used to wash and disperse the cell debris. Medium without fetal bovine serum (FBS; Thermo Scientific, Logan, UT, USA) was added to constituents with different concentrations, and the plates were then cultured in the incubator and photographed every 12 h using an inverted microscope (TE2000-U; Nikon, Tokyo, Japan). The migration and cell movement throughout the wound area were examined and calculated using the free software TScratch [42]. Magnification: 100 Bars, SD.

### 3.4. Separate Lymphocytes in Peripheral Blood in Humans

After fresh human blood (the donor is the author of this research) and PBS (pH 7.0) were diluted and thoroughly mixed, the mixture was slowly placed in Ficoll-Hypaque (Sigma Chemical Co.). The recycled lymphocytes remaining after centrifugation were cleansed with PBS.

### 3.5. Cytokine Antibody Array Assay

Linalool (227 μM) and *p*-coumaric acid (478 μM) were added into lymphocytes, respectively, and cultivated in incubators at a temperature of 37 °C with 5% CO_2_ for 24 h. Supernatant was retained after centrifugation for later testing. Blocking buffer was added into the prepared membrane (RayBio^®^Human Cytokine Antibody Array, Norcross, GA, USA) and removed after 30 min at room temperature. Then, the supernatant waiting to be tested was added and removed after 2 h of shaking at 37 °C. Wash buffer I was added and shaken for 5 min at room temperature; this method was repeated 3 times before removal. Wash buffer II was added and shaken for 5 min at room temperature; this method was repeated twice before removal. After removal, 1 mL 1× biotin-conjugated anti-cytokine was added and shaken for 2 h at room temperature before removal. Wash buffer I was added and shaken for 5 min at room temperature; this method was repeated 3 times before removal. Wash buffer II was added and shaken for 5 min at room temperature; this method was repeated twice before removal. After removal, 2 mL 1× streptavidin-HRP was added and shaken for 2 h at room temperature before removal. 2 mL wash buffer I was added and shaken for 5 min at room temperature; this method was repeated 3 times before removal. After removal, detection buffer was added and left to stand without sunlight for 2 min and then the image of membrane was scanned and saved using a scanner after pressing. The quantification analysis of the density for each spot on the array was measured using AlphaEaseFC software (Alpha Innotech Corp, San Leandro, CA, USA).

### 3.6. Statistical Analysis

All data were analyzed using the Statistical Package for the Social Sciences Ver. 22 software (SPSS Inc., Chicago, IL, USA). Data are the mean ± SD from at least triplicate experiment. The significance of the differences was analyzed by one-way analysis of variance (ANOVA), with *p* < 0.05 or *p* < 0.01 as considered significant.

## 4. Conclusions

The results obtained in this study demonstrate that linalool can have cytotoxic effects by inducing cells to undergo apoptosis and triggering cell death. The majority of studies pertaining to the monoterpenoid linalool are typically focused on its capacity to suppress microorganisms, with few having endeavored to research aspects of its anti-cancer activity. Linalool offers tremendous potential in terms of treating cancer and immunity. We believe that this substance can substantially enhance tumor treatments and provide novel starting points for future anti-cancer research.
